# Neuromodulatory Effect of Transcranial Direct Current Stimulation on Resting-State EEG Activity in Internet Gaming Disorder: A Randomized, Double-Blind, Sham-Controlled Parallel Group Trial

**DOI:** 10.1093/texcom/tgaa095

**Published:** 2021-01-04

**Authors:** Ji-Yoon Lee, Joon Hwan Jang, A Ruem Choi, Sun Ju Chung, Bomi Kim, Minkyung Park, Sohee Oh, Myung Hun Jung, Jung-Seok Choi

**Affiliations:** 1 Department of Psychiatry, SMG-SNU Boramae Medical Center, Seoul 07061, Republic of Korea; 2 Department of Psychiatry, Seoul National University Health Service Center, Seoul 08826, Republic of Korea; 3 Department of Medicine, Seoul National University College of Medicine, Seoul 03080, Republic of Korea; 4 Medical Research Collaborating Center, SMG-SNU Boramae Medical Center, Seoul 07061, Republic of Korea; 5 Department of Psychiatry, Hallym University Sacred Heart Hospital, Hallym University College of Medicine, Anyang 14068, Republic of Korea; 6 Department of Psychiatry and Behavioral Science, Seoul National University College of Medicine, Seoul 03080, Republic of Korea

**Keywords:** beta coherence, gamma power, Internet gaming disorder, resting-state EEG, tDCS

## Abstract

Transcranial direct current stimulation (tDCS) has been used as an adjunct therapy for psychiatric disorders; however, little is known about the underlying neurophysiological effects of tDCS in Internet gaming disorder (IGD). We investigated the effects of tDCS on cortical activity using resting-state electroencephalography (EEG) in patients with IGD. This randomized, double-blind, sham-controlled parallel group study of tDCS (ClinicalTrials.gov NCT03347643) included 31 IGD patients. Participants received 10 sessions (2 sessions per day for 5 consecutive days) of active repetitive tDCS (2 mA for 20 min per session) or sham stimulation. Anode/cathode electrodes were placed over the left and right dorsolateral prefrontal cortex, respectively. In total, 26 participants (active group *n* = 14; sham group *n* = 12) completed the trial. Resting-state EEG spectral activity (absolute power) and functional connectivity (coherence) were used to assess the effects of tDCS on cortical activity before stimulation and 1 month after the intervention. Active stimulation of tDCS suppressed increase of intra-hemispheric beta coherence after 1 month, which was observed in the sham group. The 1-month follow-up assessment revealed that absolute gamma power in the left parietal region was decreased in the active group relative to the sham group. Our findings suggest that repetitive tDCS stabilizes fast-wave activity in IGD.

## Introduction

The Internet has rapidly become an essential part of our daily lives. In particular, the use of Internet-based games has rapidly expanded because of their easy availability and entertainment value. However, unlimited access to the entertainment provided by Internet games may cause users to have preoccupation with gaming, spend more time gaming to satisfy the urge, and fail to reduce playing. Consequently, Internet gaming users become addicted to them. Various adverse effects of Internet gaming have been reported (Wu et al. 2018), establishing Internet gaming disorder (IGD) as a significant psychiatric problem. The Diagnostic and Statistical Manual of Mental Disorders, Fifth Edition (DSM-5) defines IGD as the repetitive use of Internet-based games resulting in preoccupation or obsession, withdrawal symptoms, and overuse and causing significant impairment or distress in several aspects of daily life (American Psychiatric Association 2013). The International Classification of Diseases 11th Revision included gaming disorder, defined as a pattern of gaming behavior (“digital-gaming” or “video-gaming”) characterized by impaired control over gaming, increasing priority given to gaming over other activities, and continuation or escalation of gaming despite the occurrence of negative consequences (World Health Organization 2018). Individuals with IGD commonly have comorbid psychiatric disorders including substance use disorder, attention-deficit hyperactivity disorder (ADHD), depression, hostility, and social anxiety disorder (Ko et al. 2012). Due to the rapid increase in the number of individuals diagnosed with IGD and its side effects, developing effective therapeutic interventions is necessary to treat patients with IGD.

Over the past few decades, neuroimaging has become increasingly important for the study of the neural correlates of IGD (Kuss and Griffiths 2012). Electroencephalography (EEG) has several advantages over other neuroimaging tools, including high temporal resolution, noninvasiveness, and significantly lower cost. Resting-state EEG is an electrophysiological recording of spontaneous electrical activity in the brain that reflects the brain state prior to information processing (Wang et al. 2013). Several studies have used EEG recordings to investigate the neural mechanisms underlying IGD. The findings revealed that absolute power was decreased in the beta band and increased in the gamma band of patients with IGD relative to values in healthy controls (Choi et al. 2013). Increased power in the gamma band is associated with impaired inhibitory control, which is a key feature of addiction and indicator of the severity of addiction in patients with IGD (Andone et al. 2016). Moreover, another study suggested decreased absolute beta power as a potential trait biomarker, consistent with previous findings (Son et al. 2015). In terms of response inhibition, an ERP Go/NoGo paradigm study showed that patients with IGD had more demand for cognitive control in the early stages of response inhibition, according to addiction severity and impulsivity (Kim et al. 2017). An EEG coherence study found that phasic synchrony in the IGD group indicated increased intra-hemispheric gamma coherence compared with the alcohol use disorder and healthy control groups. The authors suggested that the heightened phasic synchrony in the gamma band in the resting state may be an important neurophysiological marker of IGD (Park JH et al. 2017a). In a longitudinal coherence study, participants with IGD exhibited increased intra-hemispheric coherence in the beta and gamma bands at baseline. These abnormal phase synchrony patterns were not normalized after 6 months of pharmacotherapy, despite significant improvement in IGD symptoms (Park et al. 2018). Based on the findings of previous studies, distinct patterns in beta and gamma EEG activity in patients with IGD may be significant neurophysiological markers of IGD.

Several types of therapy have been used to treat IGD, including cognitive–behavioral therapy and pharmacological treatments (King and Delfabbro 2014; Andone et al. 2016). Transcranial direct current stimulation (tDCS) is a noninvasive brain stimulation technique in which a low-intensity direct current applied to the scalp modulates neuronal resting membrane potentials. In general, anodal tDCS enhances cortical excitability, and cathodal tDCS reduces cortical excitability (Nitsche et al. 2008). Few studies have investigated the therapeutic effectiveness of tDCS for IGD. A previous study of online gamers receiving tDCS using 18F-fluoro-2-deoxyglucose positron emission tomography found decreases in the weekly hours spent on gaming and in Internet addiction and depression scores, along with increased self-control scores after tDCS sessions (Lee et al. 2018). Interestingly, the abnormal right-greater-than-left asymmetry of regional cerebral glucose metabolism in the dorsolateral prefrontal cortex (DLPFC) was partially alleviated (Lee et al. 2018). The findings of tDCS studies are generally encouraging from a clinical point of view; however, the extent of tDCS-mediated effects on brain physiology requires further investigation. Taken together, the previous studies reported that patients with IGD showed higher impulsivity (Lee et al. 2012; Choi et al. 2014), frontal lobe dysfunctions (Choi et al. 2013; Son et al. 2015), and difficulties in response inhibition (Kim et al. 2017). Those neurophysiological features were related with the mechanism of IGD.

Therefore, we assessed whether repetitive bilateral tDCS over the DLPFC would change those neurophysiological features of IGD over a defined time course. In particular, the effectiveness of tDCS in patients with IGD has not been investigated in a randomized, double-blind, sham-controlled study. Based on previous studies (Choi et al. 2013; Son et al. 2015; Park JH et al. 2017a; Park et al. 2018), we hypothesized that repetitive tDCS over the DLPFC would affect fast-frequency EEG activity (spectral activity and coherence) differently than sham stimulation would in patients with IGD. To our knowledge, no studies have investigated the short-term effects of tDCS on cortical activity using resting-state EEG in patients with IGD.

## Materials and Methods

### Trial Design

This clinical trial was a single-center, double-blind, randomized, sham-controlled parallel group trial.

### Participants

Thirty-one adult males participated in this study. Participants ranged in age from 18 to 34 years and were seeking treatment for problems related to excessive Internet gaming. Diagnosis of IGD and exclusion of comorbid psychiatric disorders were made by a clinically experienced psychiatrist based on the criteria of the DSM-5. The Mini-International Neuropsychiatric Interview (Lecrubier et al. 1997) was administered to identify past and current psychiatric illnesses of participants. In order to investigate neuromodulatory effects of tDCS in pure IGD patients without comorbid psychiatric disorders, the included participants had no comorbid psychiatric diagnoses including ADHD, substance abuse or dependence, and depressive or anxiety disorders, and had no history of head injury or cognitive delay. All participants were medication-naive at the time of assessment and during the tDCS intervention. The Korean version of the Wechsler Adult Intelligence Scale-IV (WAIS-IV) was administered to all subjects to estimate intelligence quotient and cognitive delay, and only subjects with WAIS-IV scores >80 were included in the study. Three participants dropped out before the randomization procedure, and 2 dropped out after the brain stimulation intervention; thus, 26 of 31 (83.9%) participants successfully completed the study (active stimulation group, *N* = 14; sham stimulation group, *N* = 12). The research was fully explained, and all participants provided written informed consent prior to participation in the study. The participants received a monetary reward of about $50 US dollars for participation in the study. This clinical trial was reported according to CONSORT guidelines and was registered under ClinicalTrials.gov (identifier NCT03347643). The study was conducted in accordance with the Declaration of Helsinki and was approved by the Institutional Review Board of SMG-SNU Boramae Medical Center, Seoul, Republic of Korea.

### Procedures

**Figure 1:**
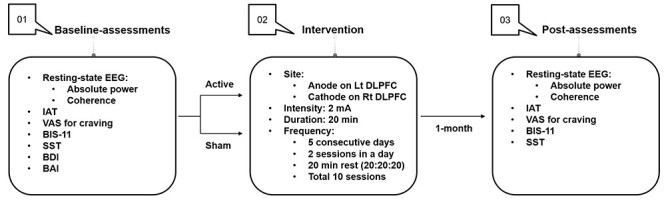
Diagram of the general procedure. Lt, left; Rt, right.

Baseline assessments included resting-state EEG as primary outcomes and clinical status (Young’s Internet Addiction Test [IAT] and craving for Internet gaming), psychological and neurocognitive measures of impulsivity (Barratt Impulsiveness Scale-11 [BIS-11]) and response inhibition (the stop-signal task [SST]), and mood status including depressive (Beck Depression Inventory-II [BDI-II]) and anxiety (Beck Anxiety Inventory [BAI]) symptoms as secondary outcomes. After randomization, participants underwent 10 sessions (2 sessions per day for 5 consecutive days) of tDCS. After repeated tDCS, resting-state EEG measures, clinical status, and psychological and neurocognitive measures of impulsivity and response inhibition were administered (Fig. 1).

### Randomization

Participants were randomly assigned to the active stimulation group (active) or the sham stimulation group (sham) in a 1:1 ratio using a randomization list created in SPSS version 20 (IBM Corp., Armonk, NY) with block randomization (block size four). The stimulation devices were preprogramed to administer active or sham stimulation according to the randomization list code. To ensure double blinding, the investigator did not have access to this list during the study. Participants’ adherence to the inclusion and exclusion criteria was verified before the randomization procedure was performed. Investigators and patients were blinded to the treatment assignments. Treatment algorithms were determined by the study statistician. A comprehensive document describing the randomization procedure is kept confidentially in the SMG-SNU Boramae Medical Center, Seoul, Republic of Korea.

### Intervention

For tDCS, the anode electrode was placed over the left DLPFC (F3) and the cathode over the right DLPFC (F4) according to the 10-20 International system. For each daily session, the current flowed continuously during two 20-min stimulation periods (2.0 mA) separated by a 20-min rest interval (no stimulation; a 20:20:20 schedule). This protocol was based on that of a previous study, which found extended effects of tDCS (Monte-Silva et al. 2013). In the sham tDCS group, the electrodes were placed in the same positions, but the device was turned off after the current was ramped up (30 s) and down (30 s). In this way, the participants remained blinded to the respective stimulation condition, as many individuals experience an itching sensation initially during stimulation (Brunoni et al. 2011). After the baseline visit, the participants received 10 active or sham sessions (2 sessions per day for 5 consecutive days) using the tDCS device (Ybrain, Seongnam, South Korea). The participants were asked to report any adverse effects after each session.

### Measurements

#### E‌EG Recording for Primary Measures

The participants were seated in a resting position in an isolated sound-shielded room connected to the recording room via a one-way glass window. EEG was recorded for 10 min: 4 min with eyes closed, 2 min with eyes open, and 4 min with eyes closed. EEG activity was recorded using a 64-channel Quik-Cap (Compumedics Neuroscan, El Paso, TX) in accordance with the modified International 10-20 system, in conjunction with recordings from vertical and horizontal electrooculograms and one bipolar reference electrode connected to the mastoid. All EEG recordings were obtained using SynAmps 2 (Compumedics, Abbotsford, Victoria, Australia) and the Neuroscan system (Scan 4.5; Compumedics). EEG signals were amplified at a sampling rate of 1000 Hz using a 0.1–100 Hz online bandpass filter and a 0.1–50 Hz offline bandpass filter; the electrode impedance was kept below 5 kΩ.

All acquired EEG data were processed using NeuroGuide software (ver. 2.6.1; Applied Neuroscience, St. Petersburg, FL). Previous study reported that the linked ear (LE) reference is suitable for coherence analyses compared with an average reference and the Laplacian reference (Thatcher et al. 2004). For the analyses, 19 of the 64 channels were selected according to a montage set with LE references from the NeuroGuide as follows: FP1, F3, F7, Fz, FP2, F4, F8, T3, C3, Cz, T4, C4, T5, P3, O1, Pz, T6, P4, and O2. All EEG recordings obtained under the eyes-closed conditions were selected, and artifact removal was performed offline using the artifact rejection toolbox in the NeuroGuide software. Additionally, EEG recordings were visually inspected to eliminate eye muscle movements and other artifacts. Artifact-free epochs under the eyes-closed conditions were selected for spectral and coherence analyses. The accepted EEG epochs with absolute (uV^2^) data were smoothed using fast Fourier transforms and averaged over 5 frequency bands using the NeuroGuide spectral analysis system: delta (1–4 Hz), theta (4–8 Hz), alpha (8–12 Hz), beta (12–30 Hz), and gamma (30–40 Hz). Coherence values were calculated for all pairwise combinations of the 19 channels for each of the 5 frequency bands using NeuroGuide software. The coherence was calculated using a previously defined method (Thatcher et al. 2008; Park SM et al. 2017b). The following equation was used to determine coherence.

$$\begin{eqnarray*} \mathrm{Coherence}\ (f) &&=\left(\Sigma \mathrm{N}\left(a(x)u(y)+b(x)v(y)\right)\right)2\nonumber\\ &&\quad+\,\left(\Sigma \mathrm{N}\left(a(x)v(y)+b(x)u(y)\right)\right)2/\nonumber\\ &&\quad\times\,\Sigma \mathrm{N}\left(a(x)2+b(x)2\right)\ \Sigma \mathrm{N}\ \left(u(y)2+v(y)2\right) \end{eqnarray*}$$

Where$a(x)=\mathrm{cosine}\ \mathrm{coefficent}\ \mathrm{for}\ \mathrm{the}\ \mathrm{frequency}\ (f)\ \mathrm{for}\ \mathrm{channel}\ x$, $b(x)=\mathrm{sine}\ \mathrm{coefficent}\ \mathrm{for}\ \mathrm{the}\ \mathrm{frequency}\ (f)\ \mathrm{for}\ \mathrm{channel}\ x$, $u(y)=\mathrm{cosine}\ \mathrm{coefficent}\ \mathrm{for}\ \mathrm{the}\ \mathrm{frequency}\ (f)\ \mathrm{for}\ \mathrm{channel}\ y$, and $v(y)=\mathrm{sine}\ \mathrm{coefficent}\ \mathrm{for}\ \mathrm{the}\ \mathrm{frequency}\ (f)\ \mathrm{for}\ \mathrm{channel}\ y$.

In total, 171 intra-hemispheric and inter-hemispheric pairwise combinations of electrodes were obtained, and the intra-hemispheric coherence was calculated for the F3–C3, F3–T3, F3–P3, C3–T3, C3–P3, and T3–P3 electrode pairs in the left hemisphere and the F4–C4, F4–T4, F4–P4, C4–T4, C4–P4, and T4–P4 electrode pairs in the right hemisphere. Inter-hemispheric coherence was calculated for the F3–F4, C3–C4, T3–T4, and P3–P4 electrode pairs.

#### Clinical and Neurocognitive Assessments for Secondary Measures

After acquisition of resting-state EEG data, clinical and neurocognitive assessments were performed as follows.

##### Young’s Internet Addiction Test

The severity of IGD was assessed using Young’s IAT (Young 1998; Beard and Wolf 2001), which includes 20 items rated using 5-point scales with possible total scores ranging from 20 to 100. The Cronbach’s alpha coefficient of IAT was 0.914.

##### Craving for gaming

Craving for gaming was assessed using a Visual Analog Scale (VAS). Participants were instructed to rate their caving to play game on each session from 0 (none) to 10 (maximum).

##### Barratt Impulsiveness Scale-11

The BIS-11 assesses impulsivity based on three subscales: cognitive impulsiveness (e.g., “I get easily bored when solving thought problems”), motor impulsiveness (e.g., “I do things without thinking”), and nonplanning impulsiveness (e.g., “I am more interested in the present than in the future”) (Patton et al. 1995). The Cronbach’s alpha coefficient of BIS-11 was 0.878.

##### Beck Depression Inventory-II

The BDI-II includes 21 items that measure the severity of depressive symptoms during the past 2 weeks (Beck et al. 1996). Items are scored on a 4-point Likert scale from 0 to 3, with total scores ranging from 0 to 63; higher scores indicate more severe depressive symptoms. The Cronbach’s alpha coefficient of BDI-II was 0.933.

##### Beck Anxiety Inventory

The BAI is a 21-question, multiple choice self-report inventory used to measure an individual’s level of anxiety during the last week, focusing primarily on somatic symptoms. The items are scored on a 4-point Likert scale ranging from 0 (not at all) to 3 (severely: “It bothered me a lot”; Beck et al. 1988). The scores of the 21 items are summed to yield a single anxiety score. The Cronbach’s alpha of BAI was 0.910.

##### Stop-signal task

The SST is a neurocognitive test used to assess the ability to inhibit prepotent responses. Participants were instructed to touch a press-pad as quickly and accurately as possible when an image of an arrow was shown, but to avoid hitting the pad when a beep sound accompanied the arrow. The SST was selected from the Cambridge Neuropsychological Test Automated Battery (CANTAB, http://www/camcog.com, Cambridge Cognition, Ltd, Cambridge, UK) and run on an Acorn BBC Master 128 microcomputer with a high-resolution Microvitec (Acorn Computers, Ltd, Cambridge, UK) 12-inch video display unit and a Microvitec Touchtec 501 touch-sensitive screen. Participants sat at a comfortable height ~0.5 m from the monitor.

### Statistical Analysis

Demographic and clinical variables were compared between the active and sham groups using Student’s *t*-tests, and group comparisons before and after the tDCS intervention were performed using generalized estimating equation (GEE). GEEs are used to estimate possible unknown correlations between repeated or multiple outcomes in the same subject. The absolute power at the 19 electrodes was divided into 9 sites according to brain region and hemisphere and averaged as follows (Barry et al. 2010): left frontal (Fp1, F3, and F7), midline frontal (Fz), right frontal (Fp2, F4, and F8), left central (T3 and C3), midline central (Cz), right central (T4 and C4), left posterior (T5, P3, and O1), midline posterior (Pz), and right posterior (T6, P4, and O2). Each EEG analysis included 9 sites to reflect region (frontal, central, and posterior) and hemisphere (left, midline, and right). Next, a GEE was used to assess EEG characteristics in each band. For the absolute power analysis, group (active and sham), region (frontal, central, and posterior), hemisphere (left and right), and their interaction effects were tested in each band using a GEE. In the coherence analysis, intra-hemispheric and inter-hemispheric coherence values were assessed according to intra-hemispheric coherence: group (active and sham) × region (frontocentral, frontotemporal, frontoparietal, centrotemporal, centroparietal, and temporoparietal) × hemisphere (left and right) and inter-hemispheric coherence: group (active and sham) × region (frontal, central, temporal, parietal). Bonferroni-corrected post hoc comparisons were performed to determine specific between-group differences (*P* < 0.025). Furthermore, Spearman’s correlation analyses were performed to determine the relationships between clinical and EEG features that showed significant main or interaction effects in the GEE analyses. All statistical tests were performed using SPSS software (version 23.0, IBM Corp., Armonk, NY).

## Results

### Demographic and Clinical Data

The demographic and clinical characteristics of the active and sham groups are shown in Table 1. The demographic and clinical characteristics were not significantly different between the active and sham groups at baseline. The baseline and 1-month follow-up scores on the IAT, VAS (craving), BIS-11, and SST did not differ between groups. Cohen’s *d* effect size is shown in Table 1. Specific descriptive statistics of clinical characteristics were described in Supplementary Table S1.

**Table 1:** Demographic and clinical characteristics between the active and sham groups

	Active group (*n* = 14) Mean ± SD		*Sham group (*n* = 12) Mean ± S.D*		t	*P*	Cohen’s *d*
Demographic data						
Age	23.071 ± 5.784		25.333 ± 8.937		−0.777	0.445	0.301	
Education (years)	12.857 ± 1.748		12.750 ± 2.006		0.146	0.885	0.057	
Game usage in weekday (h)	5.464 ± 4.116		2.625 ± 2.797		2.020	0.055	0.807	
Game usage in weekend (h)	6.286 ± 4.237		8.375 ± 18.070		−0.421	0.678	0.159	
Clinical features						
	Baseline	1-month follow-up	Baseline	1-month follow-up	Wald ${x}^2$*/t*	*P*
IAT	61.929 ± 14.897	55.071 ± 15.405	64.000 ± 14.954	62.500 ± 18.720	1.274	0.259
Craving (VAS)	6.786 ± 1.424	5.286 ± 1.978	7.167 ± 1.337	6.083 ± 1.424	2.562	0.109
BIS-11	68.714 ± 8.660	67.357 ± 8.608	69.833 ± 13.381	70.417 ± 9.462	0.611	0.434
SST total errors	1.786 ± 1.847	1.929 ± 4.376	2.583 ± 5.035	2.667 ± 5.549	0.228	0.633
SST proportion of successful stop in last half trials	0.500 ± 0.120	0.513 ± 0.098	0.525 ± 0.139	0.473 ± 0.136	0.067	0.795
BDI	23.500 ± 13.944	NA	23.083 ± 9.986	NA	0.086	0.932
BAI	19.000 ± 12.070	NA	18.250 ± 12.983	NA	0.153	0.880

**Note**. Group comparisons of clinical features before and after the tDCS intervention were performed using GEE. SD, standard deviation.

### E‌EG Activity

**Figure 2:**
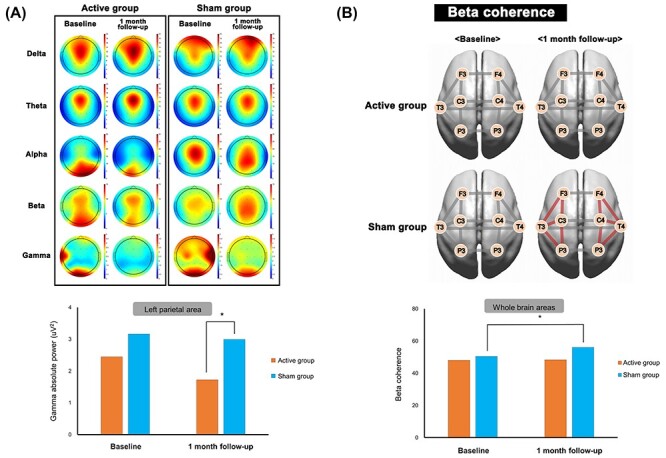
Neuromodulatory effects of tDCS in the absolute power (*A*) and coherence (*B*) before and after 10 sessions for 5 consecutive days in the active and sham groups. Red line in (*B*) represents increased beta coherence at 1-month follow-up after repetitive tDCS compared with baseline in the sham group.

The absolute power analysis revealed a significant group × time × hemisphere interaction effect for the gamma band (Table 2 and Fig. 2). The baseline absolute gamma value was not significantly different between the active and sham groups; however, at 1 month after the intervention, the absolute gamma power in the left parietal area was lower in the active group than in the sham group (*P* = 0.016). We found group effects in terms of delta, theta, alpha, and beta power; however, the Bonferroni post hoc test showed no significant differences between the groups.

**Table 2:** Model effects for absolute power between the active and sham groups

Absolute power	Wald ${x}^2$	df	*P*	Post hoc
Delta				
Group	0.427	1	0.513	
Time	1.616	1	0.204	
Region	307.382^***^	8	<0.001	N.S.
Group × Time	0.001	1	0.979	
Group × Time × Region	13.341	8	0.101	
Theta				
Group	0.026	1	0.871	
Time	0.141	1	0.707	
Region	211.825^***^	8	<0.001	N.S.
Group × Time	0.017	1	0.896	
Group × Time × Region	5.949	8	0.653	
Alpha				
Group	1.315	1	0.251	
Time	0.217	1	0.641	
Region	64.258^***^	8	<0.001	N.S.
Group × Time	0.364	1	0.546	
Group × Time × Region	10.896	8	0.208	
Beta				
Group	0.004	1	0.951	
Time	0.026	1	0.871	
Region	147.764^***^	8	<0.001	N.S.
Group × Time	1.135	1	0.287	
Group × Time × Region	35.919^***^	8	<0.001	N.S.
Gamma				
Group	2.427	1	0.119	
Time	0.994	1	0.319	
Region	46.283^***^	8	<0.001	N.S.
Group × Time	0.178	1	0.673	
Group × Time × Region	19.042^*^	8	0.015	1 m: Left parietal: active < sham

Note: N.S., not significant. The Bonferroni-corrected post hoc comparison was used (*P* < 0.025).

^*^ *P* < 0.05.

^**^ *P* < 0.01.

^***^ *P* < 0.001.

The intra-hemispheric coherence data are shown in Table 3 and Fig. 2. We found group effects for the delta (*P* < 0.001), theta (*P* < 0.001), alpha (*P* < 0.001), beta (*P* < 0.001), and gamma (*P* < 0.001) bands, indicating decreased intra-hemispheric coherence in all frequency bands in the active group relative to the sham group. Furthermore, we found a significant group × time effect in intra-hemispheric beta coherence. In the sham group, the intra-hemispheric beta coherence values were significantly higher at the 1-month follow-up than at baseline, whereas those values did not differ in the active group (post hoc Bonferroni correction, *P* < 0.001). Inter-hemispheric coherence showed no significant group, group × time, or group × time × region interaction effects (Supplementary Table S2).

**Table 3:** Model effects for intra-hemispheric coherence between the active and sham groups

Intra-hemispheric coherence	Wald ${x}^2$	df	*P*	Post hoc
Delta				
Group	21.750^***^	1	<0.001	Active < sham
Time	0.001	1	0.975	
Region	1063.323^***^	5	<0.001	N.S.
Hemisphere	0.034	1	0.854	
Group × Time	2.528	1	0.112	
Group × Time × Region	3.255	5	0.661	
Group × Time × Hemisphere	0.400	1	0.527	
Group × Time × Region × Hemisphere	0.307	5	0.998	
Theta				
Group	23.272^***^	1	<0.001	Active < sham
Time	0.022	1	0.881	
Region	1182.045^***^	5	<0.001	N.S.
Hemisphere	0.100	1	0.752	
Group × Time	3.846	1	0.050	
Group × Time × Region	2.585	5	0.764	
Group × Time × Hemisphere	0.916	1	0.339	
Group × Time × Region × Hemisphere	0.673	5	0.984	
Alpha				
Group	31.810^***^	1	<0.001	Active < sham
Time	1.051	1	0.305	
Region	827.248^***^	5	<0.001	N.S.
Hemisphere	0.168	1	0.682	
Group × Time	2.021	1	0.155	
Group × Time × Region	0.517	5	0.991	
Group × Time × Hemisphere	0.022	1	0.881	
Group × Time × Region × Hemisphere	0.579	5	0.989	
Beta				
Group	20.133^***^	1	<0.001	Active < sham
Time	6.657	1	0.010	
Region	577.022^***^	5	<0.001	N.S.
Hemisphere	1.460	1	0.227	
Group × Time	5.561^*^	1	0.018	Sham: baseline <1 m
Group × Time × Region	0.713	5	0.982	
Group × Time × Hemisphere	0.061	1	0.806	
Group × Time × Region × Hemisphere	0.597	5	0.988	
Gamma				
Group	14.366^***^	1	<0.001	Active < sham
Time	3.916	1	0.048	
Region	138.671^***^	5	<0.001	N.S.
Hemisphere	0.914	1	0.339	
Group × Time	1.788	1	0.181	
Group × Time × Region	1.172	5	0.948	
Group × Time × Hemisphere	0.141	1	0.708	
Group × Time × Region × Hemisphere	0.165	5	0.999	

Note: The Bonferroni-corrected post hoc comparison was used (*P* < 0.025). N.S., not significant.

^*^ *P* < 0.05.

^***^ *P* < 0.001.

### Correlation Analysis

Given the significant group differences revealed by the GEE analysis, we used Spearman’s correlation analysis to assess the relationships of changes in gamma absolute power in the left parietal area and beta coherence with clinical variables. No significant correlations were found between EEG activity and clinical variables in the active or sham group.

## Discussion

Our primary goal was to investigate the short-term effects of tDCS on neurophysiological activity in patients with IGD using resting-state EEG spectral and functional connectivity analyses. We found distinct changes in gamma absolute power and beta coherence after 10 sessions of repetitive tDCS. However, the intervention had no significant effect on clinical and neurocognitive measures, including the severity of addiction, craving for gaming, impulsiveness, and response inhibition. To our knowledge, this is the first study to investigate the neuromodulatory effects of tDCS on resting-state EEG activity in patients with IGD using a randomized, double-blind, sham-controlled design.

Previous studies have found aberrant resting-state EEG activity in the beta and gamma bands in patients with IGD (Choi et al. 2013; Son et al. 2015; Park JH et al. 2017a). Gamma activity is thought to reflect a variety of neural functions, including the distribution of attentional resources, feature binding, and response inhibition (Müller et al. 2000; Debener et al. 2003; Tallon-Baudry 2003; Tallon-Baudry et al. 2005; Barry et al. 2010; van Wingerden et al. 2010). Increased gamma-band activity during the resting state is associated with impaired inhibitory control and trait impulsivity and with addiction severity in patients with IGD, suggesting that increased gamma activity reflects disorganized neuronal activity in the resting state (Choi et al. 2013). We found that repetitive anodal stimulation of the left DLPFC decreased gamma absolute power in the left parietal cortex in patients with IGD relative to that in the sham group. This finding indicates that tDCS may have a neurophysiological effect by modulating gamma activity associated with inhibitory control, a key feature of addiction, for up to 1 month. Furthermore, we found that the effects of tDCS on the DLPFC extended to other brain regions including the parietal cortex. Michels et al. (2013) reported that information flow involved both the parietal and frontal regions in eyes-closed resting-state EEG.

We found a significant group × time effect (*x*^2^ = 5.561, *P* = 0.018) for intra-hemispheric beta coherence. In the sham group, the 1-month intra-hemispheric beta coherence value was significantly higher than that the baseline, while the post hoc test revealed no difference between baseline and 1-month intra-hemispheric beta coherence in the active group, suggesting that active stimulation of tDCS suppressed increase of intra-hemispheric beta coherence after 1 month, which was observed in the sham group. Overall, our findings suggest that tDCS of the DLPFC may inhibit the dysfunctional changes in beta coherence associated with IGD. Increased beta coherence is an electrophysiological marker of hyperexcitability caused by an excitation–inhibition imbalance in the brain (Rangaswamy et al. 2002; Begleiter and Porjesz 2006) and is a risk factor for IGD (Park JH et al. 2017a; Youh et al. 2017; Park et al. 2018). Park et al. (2018) reported that increased beta coherence was sustained for 6 months during the pharmacological treatment, suggesting that increased beta coherence should be considered a potential trait marker of IGD rather than a state marker. However, we found that repetitive tDCS stabilized beta coherence, suggesting that repetitive tDCS of the DLPFC induces neuromodulatory changes in brain connectivity as measured by beta coherence in patients with IGD and may also influence interactions between interconnected brain regions beyond the targeted area (Sandrini et al. 2020).

Taken together, as our hypotheses based on the previous studies showing aberrant EEG activity in the beta and gamma frequencies associated with IGD, our findings suggest that repetitive tDCS of 10 sessions for 5 consecutive days stabilized fast-wave activity as measured by gamma absolute power and beta coherence in patients with IGD. As a rule, delta-, theta-, and alpha-band activity is associated with cortical idling, whereas neurophysiological signals in the beta and gamma bands are correlated with cortical processing (Başar et al. 2001; Barry et al. 2007). Therefore, we speculate that the stabilized fast-wave activity induced by repetitive tDCS influences cognitive functions such as response inhibition, inhibitory control, and addictive behaviors associated with IGD. Recently, Sandrini et al. (2020) reported that tDCS facilitated response inhibition by modulating neural activity and functional connectivity in an integral part of the response inhibition network. However, tDCS did not significantly alter response inhibition as measured by SST in our study. Longer follow-up studies with larger samples are needed to confirm the effects of tDCS on neurocognitive functioning and clinical symptoms in patients with IGD.

There are other issues which could be considered in the present findings. Although patients with IGD did not meet diagnostic criteria of comorbid depressive or anxiety disorders, they showed depressive or anxiety symptoms. However, no correlations were found between baseline depressive or anxiety symptoms and changes in EEG parameters in the active stimulation group, indicating that the neuromodulatory effects of tDCS may not be related to mood status in patients with IGD. No patients experienced adverse events during tDCS, suggesting that tDCS is safe and may be a potential treatment for patients with IGD. The duration of tDCS effects is a critical issue because the after-effects may last for minutes or hours depending on the intensity and duration of the stimulation (Mangia et al. 2014). We found that the neuromodulatory effect of tDCS persisted for at least 1 month in patients with IGD and was present before clinical changes were observed. Further study is needed to investigate the long-term neuromodulatory effects of tDCS.

Our study has several limitations. First, our sample size was relatively small and was restricted to male participants. Second, although comorbid psychiatric conditions, such as ADHD and depressive disorders, are common in patients with IGD, we excluded patients with comorbidities; thus, our sample may not be fully representative of this population. Future studies are needed to investigate the associations between tDCS-induced neuromodulatory changes and psychiatric comorbid conditions in patients with IGD. Third, a 1-month follow-up period may be too short to adequately assess the clinical effects of tDCS, including changes in the severity of addiction and long-term neuromodulating effects. Fourth, the construct validity of VAS may be low since a single question has been used for assessing craving to play game. Despite these limitations, this is the first exploratory study on the neuromodulating effects of tDCS on resting-state EEG activities and can be used to design larger confirmatory studies in IGD.

In summary, we found that repetitive tDCS of the DLPFC changed resting-state fast-wave activity in patients with IGD. Anodal stimulation of the left DLPFC reduced gamma absolute power in the left parietal cortex relative to sham stimulation in patients with IGD and stabilized beta coherence. EEG features including spectral activity as measured by absolute power and functional connectivity measured by coherence may be important neurophysiological markers of the neuroplastic response to tDCS and may be useful for the development of targeted tDCS treatment for IGD. However, the results should be treated with caution, mainly due to the small sample size in each group. Furthermore, we did not observe statistically significant changes in clinical variables or neurocognitive functioning after a 1-month follow-up period. Further study is needed to investigate the long-term effects of tDCS on the clinical symptoms associated with IGD.

## Supplementary Material

CONSORT_2010_Checklist_20201019_tgaa095

CONSORT_2020_Flow_Chart_20201019_tgaa095

Supplementary_Table_CCC_20201220_tgaa095

## Notes

The authors would like to thank all of the participants. *Conflict of interest*: None declared.
