# Supplementary figures and images for: Neuromodulatory Effect of Transcranial Direct Current Stimulation on Resting-State EEG Activity in Internet Gaming Disorder: A Randomized, Double-Blind, Sham-Controlled Parallel Group Trial
Source: Cereb Cortex Commun. 2021 Jan 4;2(1):tgaa095. doi: 10.1093/texcom/tgaa095 (PMC8152877; doi:10.1093/texcom/tgaa095)

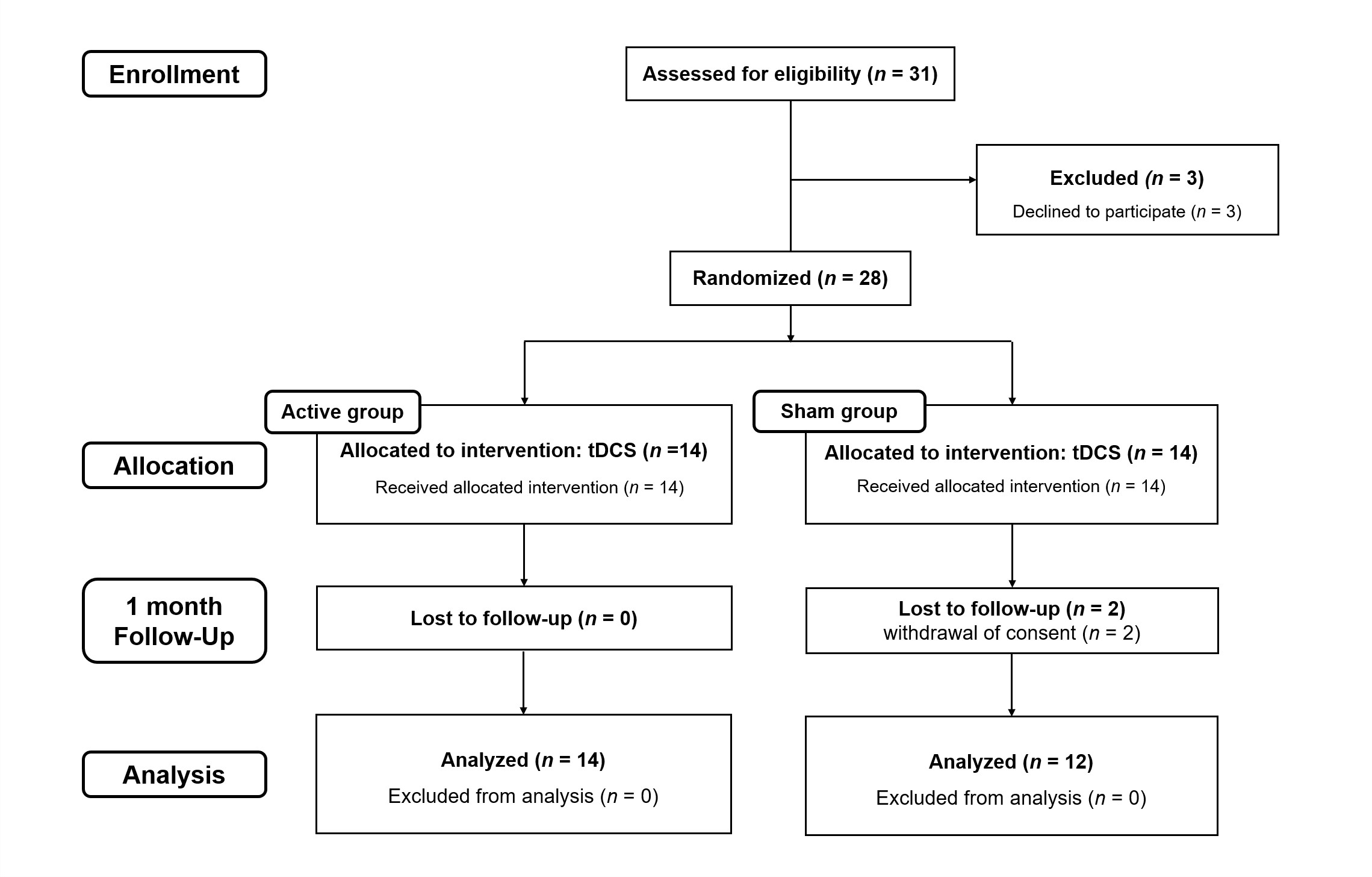

Supplement: CONSORT_2020_Flow_Chart_20201019_tgaa095 [file consort_2020_flow_chart_20201019_tgaa095.jpeg]
