# Supplementary material for: Neuromodulatory Effect of Transcranial Direct Current Stimulation on Resting-State EEG Activity in Internet Gaming Disorder: A Randomized, Double-Blind, Sham-Controlled Parallel Group Trial
Source: Cereb Cortex Commun. 2021 Jan 4;2(1):tgaa095. doi: 10.1093/texcom/tgaa095 (PMC8152877; doi:10.1093/texcom/tgaa095)
Supplement: Supplementary_Table_CCC_20201220_tgaa095 [file supplementary_table_ccc_20201220_tgaa095.docx]

**Supplementary Table S1.** Descriptive statistics of clinical characteristics

|  | **Mean** | **Standard Error** | **Range** | **Minimum** | **Maximum** | **Skewness** | **Kurtosis** |
| --- | --- | --- | --- | --- | --- | --- | --- |
| IAT | 62.885 | 2.875 | 60.0 | 37.0 | 97.0 | 0.325 | -0.139 |
| BIS-11 | 69.231 | 2.131 | 41.0 | 54.0 | 95.0 | 0.993 | 0.400 |
| SST total errors | 2.154 | 0.710 | 18.0 | 0.0 | 18.0 | 3.629 | 15.455 |
| SST proportion of successful stop in last half trials | 0.5115 | 0.025 | 0.575 | .250 | 0.825 | 0.179 | 0.925 |
| BDI | 23.346 | 2.189 | 53.00 | 2.00 | 55.00 | 0.477 | 1.846 |
| BAI | 19.462 | 2.364 | 39.00 | 4.00 | 43.00 | 0.393 | -1.264 |

**Note.** IAT = Young’s Internet addiction test; BDI = Beck depression inventory-Ⅱ; BAI = Beck anxiety inventory; BIS-11 = Barratt impulsiveness scale-11; SST = Stop Signal Task.

**Supplementary Table S2.** Model effects for inter-hemispheric coherence between the active and sham groups

| **Inter-hemispheric coherence** | **Wald** $\boldsymbol{x}^{\mathbf{2}}$ | **df** | ***P*** | ***Post hoc.*** |
| --- | --- | --- | --- | --- |
| **Delta** |  |  |  |  |
| Group | 0.316 | 1 | 0.574 |  |
| Time | 0.794 | 1 | 0.373 |  |
| Region | 977.078*** | 3 | <.001 | N.S |
| Group × Time | 0.394 | 1 | 0.530 |  |
| Group × Time × Region | 0.624 | 3 | 0.891 |  |
| **Theta** |  |  |  |  |
| Group | 1.123 | 1 | 0.289 |  |
| Time | 0.000 | 1 | 0.988 |  |
| Region | 1410.616*** | 3 | <0.001 | N.S |
| Group × Time | 1.090 | 1 | 0.296 |  |
| Group × Time × Region | 0.680 | 3 | 0.878 |  |
| **Alpha** |  |  |  |  |
| Group | 2.353 | 1 | 0.125 |  |
| Time | 0.000 | 1 | 0.995 |  |
| Region | 748.929*** | 3 | <0.001 | N.S |
| Group × Time | 0.844 | 1 | 0.358 |  |
| Group × Time × Region | 0.044 | 3 | 0.998 |  |
| **Beta** |  |  |  |  |
| Group | 5.902 | 1 | 0.015 |  |
| Time | 3.345 | 1 | 0.067 |  |
| Region | 505.446*** | 3 | <0.001 | N.S |
| Group × Time | 1.423 | 1 | 0.233 |  |
| Group × Time × Region | 0.032 | 3 | 0.998 |  |
| **Gamma** |  |  |  |  |
| Group | 7.135** | 1 | 0.008 | N.S |
| Time | 2.537 | 1 | 0.111 |  |
| Region | 150.798*** | 3 | <0.001 | N.S |
| Group × Time | 0.491 | 1 | 0.484 |  |
| Group × Time × Region | 0.272 | 3 | 0.965 |  |

**Note.** P < 0.05*, P < 0.01**, P < 0.001***; N.S = not significant; The Bonferroni-corrected post hoc comparison was used (P < 0.025).
